# Rapid chest compression effects on intracranial pressure in patients with acute cerebral injury

**DOI:** 10.1186/s13063-022-06189-w

**Published:** 2022-04-15

**Authors:** Ricardo Miguel Rodrigues-Gomes, Joan-Daniel Martí, Rosa Martínez Rolán, Miguel Gelabert-González

**Affiliations:** 1 Hospital Álvaro Cunqueiro, Vigo, Spain; 2 Universidade Santiago de Compostela, Santiago de Compostela, Spain; 3 Hospital Clinic, Barcelona, Spain

**Keywords:** Chest physiotherapy, Intracranial pressure, Rapid chest compression technique

## Abstract

**Background:**

Patients with acute brain injury often require invasive mechanical ventilation, increasing the risk of developing complications such as respiratory secretions retention. Rapid chest compression is a manual chest physiotherapy technique that aims to improve clearance of secretions in these patients. However, the rapid chest compression technique has been suggested to be associated with increased intracranial pressure in patients with acute brain injury. The aim of this work is to elucidate the effects of the technique on intracranial pressure in mechanically ventilated patients with acute brain injury. Furthermore, the effects of the technique in different volumes and flows recorded by the ventilator and the relationship between the pressure applied in the intervention group and the different variables will also be studied.

**Methods:**

Randomized clinical trial, double-blinded. Patients with acute brain injury on invasive mechanical ventilation > 48 h will be included and randomized in two groups. In the control group, a technique of passive hallux mobilization will be applied, and in the intervention group, it will be performed using the rapid chest compression technique. Intracranial pressure (main variable) will be collected with an intracranial pressure monitoring system placed at the lateral ventricles (Integra Camino).

**Discussion:**

The safety of chest physiotherapy techniques in patients at risk of intracranial hyperpressure is still uncertain. The aim of this study is to identify if the rapid manual chest compression technique is safe in ventilated patients with acute brain injury.

**Trial registration:**

NCT03609866. Registered on 08/01/2018.

## Administrative information

Note: the numbers in curly brackets in this protocol refer to SPIRIT checklist item numbers. The order of the items has been modified to group similar items (see http://www.equator-network.org/reporting-guidelines/spirit-2013-statement-defining-standard-protocol-items-for-clinical-trials/).

**Table:** 

Title {1}	The Rapid Chest Compression Technique effects on the Intracranial Pressure in mechanical ventilated patients with acute cerebral injury
Trial registration {2a and 2b}.	NCT03609866 Chest Physiotherapy Effects on Intracranial Pressure
Protocol version {3}	December, 2020
Funding {4}	A research grant from the Colexio de Fisioterapeutas de Galicia of 1000 Euros.
Author details {5a}	Ricardo Miguel Rodrigues-Gomes, Physiotherapist, MSc. Hospital Álvaro Cunqueiro, Vigo, Spain. Universidade de Santiago de Compostela, Spain Joan-Daniel Martí, Physiotherapist, PhD, Hospital Clinic, Barcelona, Spain Rosa Martínez Rolán, MD, PhD, Hospital Álvaro Cunqueiro, Vigo, Spain Miguel Gelabert-González, MD, PhD, Universidade Santiago de Compostela, Santiago de Compostela, Spain
Name and contact information for the trial sponsor {5b}	Not applicable
Role of sponsor {5c}	Not applicable

## Introduction

### Background and rationale {6a}

Critically ill patients on invasive mechanical ventilation (IMV) usually develop mucus retention, increasing the risk for associated morbidity [1–5]. Endotracheal intubation and inflation of the endotracheal tube cuff are associated with a drastic decrease of mucocilliar transportation (aprox. 10x) and imped an effective cough. Additionally, the semi-sitting position at 30°, which is currently recommended by international guidelines to prevent ventilator-associated pneumonia, may further hinder mucus clearance because of the gravity force in the greater caliber airways. Also, the use of sedative and relaxing drugs may inhibit cilia motility and respiratory muscles activation which increases mucus retention. Finally, associated comorbidities and recurrent respiratory infections also play an important role in the increased production of respiratory secretions [1–5].

Respiratory physiotherapy has been proposed as a potential strategy to prevent and/or reduce the retention of secretions in critical patients connected to invasive mechanical ventilation. The scientific evidence on the effectiveness of these techniques is not clearly conclusive and its safety has some related controversial. There are several publications that demonstrate the effectiveness of respiratory physiotherapy techniques in the mobilization of air volumes, expiratory flows, and respiratory secretions but still in an insufficient number so that clear evidence of effectiveness can be manifested in systematic review studies. Some of the respiratory physiotherapy techniques used in intensive care in ventilated patients try to modulate the ventilatory flows and improve the transport of mucus based on the double gas-liquid phase rationale. The use of these techniques is supported by laboratory evidence that mucus removal is enhanced when the expiratory flow is greater than the inspiratory flow. This is the case, for example, of the rapid chest compression technique, which aims to increase the expiratory flow and facilitate the transport of mucus to the exterior of the bronchial tree [1, 2].

In many studies, the effectiveness of the technique of rapid chest compression in the mobilization of secretions and peak expiratory flow is studied. Although in the work of Lima et al. (2008) [2] applied the thoracic compression technique, in one of its variants, to assess its effects in an animal model with atelectasis, using unilateral thoracic compression and concluding that the technique was not effective for the resolution of atelectasis with the mobilization of secretions. In a more recent work by Martí et al. (2013) [1], the technique of manual thoracic compression with different intensities was applied in pigs in IMV, and it was concluded that, with the rapid application of the technique, the secretions were mobilized outwards the bronchial tree. In this last study, controlled and randomized, the movement of respiratory secretions was studied by fluoroscopy in tracheostomized, sedated, and in IMV pigs [1, 2]. There are also studies that provide evidence of the technique of manual compression of the abdomen and chest in humans; Naue et al. (2011) [4] studied its effects associated with the increase of the support pressure to assess the amount of suctioned secretions without finding differences between groups. In 2014, the same authors published a randomized clinical trial in which they studied the increase in inspiratory pressure of 10 cmH20 associated with the technique of manual compression of the abdomen and chest with an increase in the amount of secretions suctioned, the volume of exhaled air, and improvement in the pulmonary dynamics. In the same 2014, Guimarães et al. [3] studied the effects of rapid thoracic compression on pulmonary mechanics, amount of secretions suctioned, and peak expiratory flow, finding significant differences in peak expiratory flow in the intervention group [3–5].

Although the effects of the different variants of the rapid chest compression technique in the mobilization of secretions, pulmonary dynamics, and expiratory flow continue to be studied, some authors devoted themselves to studying their effects in other systems and in certain pathologies in order to justify their use in specific clinical situations. This is the case of the effects of the manual rapid chest compression technique on intracranial pressure in patients with acute brain injuries. The first clinical trial on this matter was carried out by Thiesen et al. (2005) [6] where they studied the effects of some techniques of respiratory physiotherapy on intracranial pressure, among which manual thoracic compression with satisfactory results relative to the control of intracranial pressure was included; the conclusions were obtained without control group or randomization. Subsequently, Toledo et al. (2008) [7] studied the effect of vibro-compression on intracranial pressure in patients with cranio-encephalic trauma concluding that the respiratory physiotherapy maneuver did not increase intracranial pressure or cerebral perfusion pressure, this study was also performed without control group or randomization. Cerqueira-Neto et al. (2010) [8] and Cerqueira Neto et al. (2013) [9], also without a control, blind, or randomization group, studied the effects of the expiratory flow acceleration technique, one of the possible variants of the technique, in patients with traumatic brain injury, concluding that it did not significantly alter the intracranial pressure [6–9]. In a more recent study, Tomar et al. (2019) [10] in a randomized crossover trial comparing the manual chest percussion with the mechanical chest vibration concluded that the manual percussion was associated with ICP increase.

From the literature review, it can be verified that there is little scientific evidence on the safety of manual rapid chest compression in critically ill patients with acute brain injury in invasive mechanical ventilation [6–9, 11].

Patients with acute brain damage usually present respiratory complications associated with invasive mechanical ventilation and therefore could benefit from respiratory physiotherapy. However, the application of respiratory physiotherapy techniques in these patients could be associated with an increase in intracranial pressure [7–10, 12]. The maneuvers of respiratory physiotherapy can momentarily increase intrathoracic pressure, decreasing venous return and increasing intracranial pressure [6].

The intrathoracic pressure is directly related to the alveolar pressure and the manual rapid chest compression technique can increase momentarily the alveolar pressure. So, any technique that aims to increase the expiratory air flow must do it in a rapid way to avoid major intracranial pressure increases [6, 13–17].

According to the bibliography, the increase in intracranial pressure is a contraindication for the application of some techniques in patients with acute brain injury since it can cause a decrease in cerebral perfusion pressure if it is sustained over time or if it is not accompanied of a rise in the mean arterial pressure. The lowering of cerebral perfusion pressure leads to associated damage in the control mechanisms of blood supply in areas of penumbra, which will be responsible for part of the neuroplasticity in the recovery phases of acute brain injuries, with all its implications [10, 12, 18–20].

It is important to mention that different brain lesions may have different behaviors in terms of intracranial pressure and may present higher intervention risks [18].

Cerebral lesions with a hemorrhagic component imply a more aggressive control of the mean arterial pressure and intracranial pressure and may present extreme complications with punctual rises, as these pressure rises may cause an increase in hemorrhagic lesions due to fragility. The ischemic brain lesions may have an inverse behavior since decreases in mean arterial pressure or increases in intracranial pressure can lead to decreases in the cerebral perfusion pressure and make the penumbra areas suffer [18–21].

At present, there are no randomized clinical trials that have studied the effect of the manual rapid chest compression technique alone on intracranial pressure and that try to relate its behavior with the different kinds of acute brain injuries. Therefore, we believe that it is necessary to investigate the effects of the technique with the highest possible methodological rigor and quality [10–12].

We propose this study, trying to control as many variables as possible and studying the effects of a single technique, in order to provide methodologically sound conclusions.

### Objectives {7}

#### Main hypothesis

H0—The rapid chest compression technique does not produce a change in intracranial pressure in patients on invasive mechanical ventilation with severe acute brain injury.

H1—The rapid chest compression technique produces changes in intracranial pressure in patients on invasive mechanical ventilation with severe acute brain injury.

#### Objectives

##### Primary objective

To determine the effects of the rapid chest compression technique on intracranial pressure in patients on invasive mechanical ventilation with severe acute brain injury.

##### Secondary objectives

Establish a relationship between the type of brain injury and the behavior of intracranial pressure.

Establish a relationship between peak expiratory flow and behavior of intracranial pressure.

Establish a relationship between the pressure applied in the studied technique and the changes in the other dependent variables.

### Trial design {8}

Randomized controlled trial, double-blinded with a control group and intervention group.

## Methods: Participants, interventions, and outcomes

### Study setting {9}

The study protocol will be applied in the intensive care unit of the Hospital Álvaro Cunqueiro, a tertiary hospital in the southwest of Galicia; Spain.

### Eligibility criteria {10}

#### Inclusion criteria:

- Intubated patients with mechanical ventilation in Volume Controlled, Pressure Controlled and Volume Controlled Pressure Regulated modes during the 48 h prior to the application of the technique
- Severe head injury with a GCS≤8 before sedation or after the surgical procedures, when needed.
- Hemodynamic stability (MAP> 65 mmHg)
- Respiratory stability (PEEP <10cmH_2_O and FiO_2_ < 60%)
- Intracranial Pressure Stability (0 <ICP < 20 mmHg)
- RASS sedation scale of − 5 points
- Informed consent obtained

#### Exclusion criteria

- Thoracic fractures: unstable rib, sternal, clavicular, scapular, or vertebral
- Abdominal injuries that prevent the application of local manual compression
- Systemic or local changes that occur with increased abdominal volume
- Fractures or conditions in the lower right limb that contraindicate passive mobilization of first metatarsophalangeal joint

### Who will take informed consent? {26a}

This study has been approved by the Comité de Ética de la Investigación de Pontevedra-Vigo-Ourense (ethics board of the Galician public health system) with the registration number 2018/446. The informed consent will be given, if they agree, by the next of kin, to whom will be given written information about the participation in the trial. The relatives will be informed that they can withdraw their consent at any time and that they can claim to the Spanish Agency of Data Protection if their rights are not being fulfilled. As severe acute brain injuries have a relatively slow evolution, we do not foresee the patient’s consent.

### Additional consent provisions for collection and use of participant data and biological specimens {26b}

The consent document offers two options: (1) to erase the data after completion of the study or (2) to allow this data to be kept in an anonymized form for future studies.

## Interventions

### Explanation for the choice of comparators {6b}

The used comparator, passive first metatarsophalangeal joint mobilization, allows us to apply a technique that usually patients at this condition receive and that has no known effect in the intracranial pressure.

### Intervention description {11a}

Intervention group: the application of the technique will be carried out in a single moment and will be applied with the patient in supine decubitus with 30° elevation of head and trunk, the rapid chest compression technique will be applied once in the expiratory time of each 3 respiratory cycles during 5 min by a physiotherapist. Safety monitoring of heart rate, blood pressure, intracranial pressure, peripheral oxygen saturation, and cerebral perfusion pressure will be performed by the clinical team and the principal investigator. The intervention will stop if reached some of the safety values presented below or when the clinical team considers.

Control group: the technique will be performed in a single moment and will be applied with the patient in supine decubitus with 30° elevation of head and trunk. The passive mobilization technique of the right first metatarsophalangeal joint will be applied to the available range of flexion-extension, performing 15 repetitions per minute and for 5 min by a physiotherapist. Safety monitoring of heart rate, blood pressure, intracranial pressure, peripheral oxygen saturation, and cerebral perfusion pressure will be performed by the clinical team and the principal investigator. The intervention will stop if reached some of the safety values presented below or when the clinical team considers.

### Criteria for discontinuing or modifying allocated interventions {11b}

The interventions will stop if reached some of the safety values presented below or when the clinical team considers [11, 22, 23].

**Table:** 

Safety limits	
40 bpm < **Heart rate** < 120 bpm	
60 mmHg < **Mean arterial pressure** < 110 mmHg	
− 10 mmHg < **Intracranial pressure** < 30 mmHg	
90% < **Oxygen saturation**	
50 mmHg < **Cerebral perfusion pressure**	

The rescue plan is the usual at our ICU to manage the pathological increases in the ICP: open drainage if exists, increase sedation, apply relaxation medicines and hypertonic solution, this in a step-by-step approach.

### Outcomes {12}

The data about patient’s clinical situation and demographics will be collected directly from the clinical history. The main and secondary outcomes will be collected as described below:

#### Intracranial pressure

The intracranial pressure (mmHg) data will be recorded 1 h prior to the intervention, due to the change in mechanical ventilation mode, 30 min prior, 5 min prior, at the beginning of the intervention and every minute since the beginning, 5 and 30 min since the end with the continuous intracranial pressure monitoring system from Integra® Camino®.

#### Oxygen saturation, heart rate, and cerebral perfusion pressure

**Fig. 1:**
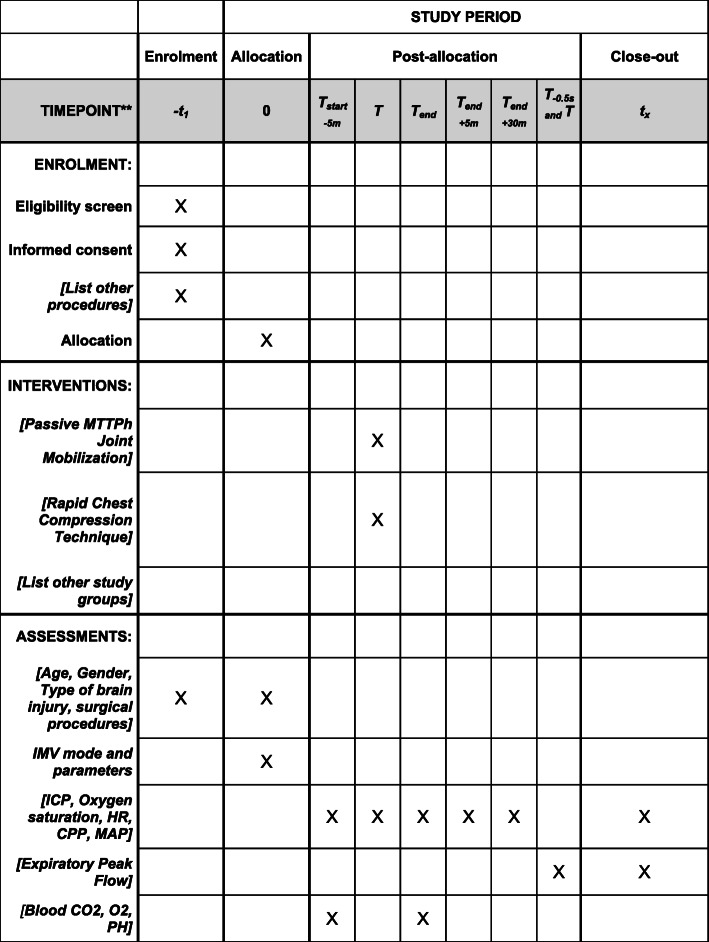
SPIRIT (Standard Protocol Items: Recommendations for Interventional Trials) table of enrollment, intervention, and assessments. *Recommended content can be displayed using various schematic formats. See SPIRIT 2013 Explanation and Elaboration for examples from protocols. **List specific timepoints in this row

The data from the 3 outcomes will be recorded from the Philips IntelliVue with the time chart as exposed in Fig. 1.

#### Expiratory and Inspiratory peak flow

Data will be collected in 30 s periods at T−60 m, T−5 m, at the beginning and every minute, T+ 5 m, and T+ 30 m; all expiratory peak flows will be checked and the best will be recorded from every 30 s sequence. Maquet Servo U and I, Hamilton C5 ventilators will be used.

#### Applied pressure

In order to assure that the technique is performed consistently, the chest compression pressure made by the Physiotherapist will be monitored and data recorded during the procedure application time. The Tekscan ComfortMat 1 will be the device used to measure and record the pressure values (g/cm^2^).

#### Blood O_2_ and CO_2_

Recent studies identify a tight control in the lower limits of blood CO_2_ partial pressure as a protective factor in case of brain injury, because high CO_2_ levels induce arterial vasodilatation as shown in laboratory studies. We will perform arterial blood gas 5 min prior to and at the end of the technique application.

#### CT scan

We will use the images acquired and interpretation made by Radiologists to identify the type and location of the injury.

### Participant timeline {13}

The participant timeline is shown in Fig. 1.

### Sample size {14}

To obtain the sample size needed for this study we worked with the software Ene 3.0 and based the estimations on the changes in intracranial pressure with the techniques performed in the study by Thiesen et al. (2005), in which the mean of baseline measures (equivalent to the control group) was 6.9 mmHg, mean in the experimental group was 7.2 mmHg 30 mins post-physiotherapy and joint standard deviation was 2.0 mmHg in patients with ICP 0–10 mm and the mean of baseline measures (equivalent to the control group) was 13.7 mmHg, mean in the experimental group was 12.7 mmHg 30 mins post-physiotherapy and joint standard deviation was 2.5 mmHg in patients with ICP 10–20 mmHg.

With a power of 80% and to detect that the difference of means is equal to the equivalence limit by means of a *T*-Student test for two independent samples, assuming that the equivalence limit is 2.0 mmHg and a level of confidence of 95%, we obtained a sample size of 25 patients in each group: control and experimental. The total number is 50 subjects for this RCT.

### Recruitment {15}

The participants included in this study will be selected from the admitted patients with severe acute brain injury, ICP monitoring, and IMV in the ICU of the Hospital Álvaro Cunqueiro, Galicia, Spain. The number of patients that we had at our ICU with severe acute brain injury in 2019 was about 80. The selected patients must meet the following inclusion criteria: patients intubated with mechanical ventilation in controlled volume mode, controlled pressure and controlled volume controlled by pressure during the 48 h prior to the application of the technique; hemodynamic stability (MAP> 65 mmHg); respiratory stability (PEEP <10cmH_2_O and FiO_2_ < 60%); stability of intracranial pressure (0 <ICP < 20 mmHg); RASS sedation scale of 5 points; and informed consent obtained (Annex 1). Patients meeting any of the exclusion criteria will not be enrolled: thoracic fractures: costal, sternal, clavicular, scapular or unstable vertebral; abdominal injuries that prevent the application of local manual compression; systemic or local changes that occur with abdominal volume increase; and fractures or conditions in the lower right limb that contraindicate the performance of passive mobilization of the 1st metatarsophalangeal joint.

## Assignment of interventions: allocation

### Sequence generation {16a}

The allocation sequence was generated at the GraphPad QuickCalcs page (https://www.graphpad.com/quickcalcs/randomize2/) with no repetition and 50 subjects with allocation in 2 groups.

### Implementation {16c}

The allocation sequence generated as described in 16a will be applied after the acceptance and informed consent signing. Once accepted to be part of the study, the PI will enroll and assign the subjects according to the sequence generated at GraphPad.

## Assignment of interventions: blinding

### Who will be blinded {17a}

*Patients* will be blinded, as they are sedated.

*Data analysts* will be blinded as the data package will be given at the end of the data collection with no reference to the main variable.

### Procedure for unblinding if needed {17b}

The need of unblinding is not permissible and if for any circumstantial reason it happens, the subject will be erased from the database and not included in this study. Another subject will be enrolled in the same group of allocation.

## Data collection and management

### Data management {19}

Data will be kept and anonymized by the principal investigator.

### Confidentiality {27}

The principal investigator will be the only person that will manage the subject’s identification information. All other persons will manage limited and anonymized information.

## Statistical methods

### Statistical methods for primary and secondary outcomes {20a}

A descriptive analysis of the collected values of the variables under study will be carried out, studying the measures of central tendency. Normal distributions will show mean and standard deviation and median and interquartile range in case of non-observation of normal distribution. The normality of the samples will be verified with the Kolmogorov-Smirnov test.

The verification of the hypothesis under study will be assessed using Student’s *T* test for independent samples in case of normality of the sample and by the *U* Mann-Whitney test otherwise.

A multiple linear regression analysis will be performed: considering as a dependent variable the intracranial pressure differences, difference or gain of expiratory flow and between the covariates, the type of technique, the pressure applied in the technique under study, the type of brain injury, blood gases changes, age, and sex.

### Interim analyses {21b}

## Oversight and monitoring

### Composition of the data monitoring committee, its role and reporting structure {21a}

DMC will not be needed due to the clinical nature of all the data.

### Plans for communicating important protocol amendments to relevant parties (e.g., trial participants, ethical committees) {25}

Protocol changes will be communicated to the Ethics Committee of Galicia and data updated at the Clinical Trials page.

### Dissemination plans {31a}

The study results and conclusions are intended to be published in 1st quartile physical therapy journals or intensive care journals.

As this is a clinical issue that should be known because it’s relevant to patients and professionals, we intend to publish it in international journals of intensive care and physiotherapy (Tables 1 and 2).

**Table 1:** Intensive care journals - 2018 Journal Impact Factor, Journal Citation Reports Science Edition (Clarivate Analytics, 2018)

Intensive care	
*American Journal of Respiratory and Critical Care Medicine*	IF: 16,494 1st Q
*Chest*	IF: 9,657 1st Q
*Intensive Care Medicine*	IF: 18,967 1st

**Table 2:** Physiotherapy journals - 2018 Journal Impact Factor, Journal Citation Reports Science Edition (Clarivate Analytics, 2018)

Physiotherapy	
*Neurorehabilitation and Neural Repair*	IF: 3,757 1st Q
*Journal of Physiotherapy*	IF: 5,551 1st Q
*Physical Therapy*	IF: 3,043 1st Q

## Discussion

Nowadays, the chest physiotherapy techniques are applied with several restrictions because of their effect in the intracranial pressure values, at least as referred to in several publications [5–12].

The main problem that we observe in almost every publication about the chest physiotherapy techniques in acute cerebral injured patients is that they usually include a protocol that has secretion suction at the end without separation of the effects. It’s known that the suction has clear reflex effects that increase the ICP and MAP even when patients are sedated, as shown in a recent study from Singh et al. (2018) [12] where they studied different drug effects on intracranial pressure, applying chest percussion and suctioning.

We believe, for our clinical experience and some publications that when adequately applied, rapid chest compression is safe and patients can benefit from its use, because invasive mechanical ventilation increases the risk of respiratory complications, making the management of acute cerebral injuries more complex and its influence is unknown on the outcomes [1–5, 10, 12].

In our trial, we intend to control the larger number of possible interactions to avoid results that can be affected by collateral techniques.

### Trial status

We will start the recruitment in January 2021, and we expect to end approximately in 1 year, because of the COVID-19 impact.

## Abbreviations

IMV: Invasive mechanical ventilation
MAP: Mean arterial pressure
PEEP: Positive end-expiratory pressure
FiO_2_: Inspired oxygen fraction
RASS: Richmond agitation and sedation scale
CT scan: Computerized tomography scan
MTTPh: Metatarsophalangeal
ICP: Intracranial pressure
HR: Heart rate
CPP: Cerebral perfusion pressure
PI: Principal investigator
DMC: Data Management Committee
IF: Impact factor
Q: Quartile
